# Multi-walled carbon nanotubes increase antibody-producing B cells in mice immunized with a tetravalent vaccine candidate for dengue virus

**DOI:** 10.1186/s12951-016-0196-7

**Published:** 2016-07-27

**Authors:** Luan P. Calegari, Roberto S. Dias, Michelle D. de Oliveira, Carine Ribeiro Pessoa, André S. de Oliveira, Ana F. C. S. Oliveira, Cynthia C. da Silva, Flavio G. Fonseca, Alice F. Versiani, Sérgio O. De Paula

**Affiliations:** 1 Federal University of Viçosa, Avenida Peter Henry Rolfs, s/n, Campus Universitário, Viçosa, MG 36570-900 Brazil; 2 Federal Institute of Education, Science and Technology of North of Minas Gerais, R. Gabriel Passos, 259, Centro, Montes Claros, MG 39400-112 Brazil; 3 Federal University of Minas Gerais, Belo Horizonte, MG Brazil

**Keywords:** Multi-walled carbon nanotubes, Vaccine, Dengue, Vero cells, Intramuscular route

## Abstract

**Background:**

In recent times, studies have demonstrated that carbon nanotubes are good candidates for use as vehicles for transfection of exogenous material into the cells. However, there are few studies evaluating the behavior of carbon nanotubes as DNA vectors and few of these studies have used multi-walled carbon nanotubes (MWCNTs) or carboxylated MWCNTs. Thus, this study aims to assess the MWCNTs’ (carboxylated or not) efficiency in the increase in expression of the tetravalent vaccine candidate (TVC) plasmid vector for dengue virus in vitro using Vero cells, and in vivo, through the intramuscular route, to evaluate the immunological response profile.

**Results:**

Multi-walled carbon nanotubes internalized by Vero cells, have been found in the cytoplasm and nucleus associated with the plasmid. However, it was not efficient to increase the messenger ribonucleic acid (mRNA) compared to the pure vaccine candidate associated with Lipofectamine^®^ 2000. The in vivo experiments showed that the use of intramuscular injection of the TVC in combination with MWCNTs reduced the immune response compared to pure TVC, in a general way, although an increase was observed in the population of the antibody-producing B cells, as compared to pure TVC.

**Conclusions:**

The results confirm the data found by other authors, which demonstrate the ability of nanotubes to penetrate target cells and reach both the cytoplasm and the cell nucleus. The cytotoxicity values are also in accordance with the literature, which range from 5 to 20 µg/mL. This has been found to be 10 µg/mL in this study. Although the expression levels are higher in cells that receive the pure TVC transfected using Lipofectamine^®^ 2000, the nanotubes show an increase in B-cells producing antibodies.

**Electronic supplementary material:**

The online version of this article (doi:10.1186/s12951-016-0196-7) contains supplementary material, which is available to authorized users.

## Background

In 2012 the World Health Organization (WHO) classified dengue as “the most important mosquito-borne disease in the world”, mainly because of the significant geographical distribution of the virus and the vector, including in areas that were previously unaffected by the disease; and also the subsequent costs brought on by dengue [1, 2]. In the same year a study conducted by Brady and colleagues suggested that a total of 3970 million people living worldwide in 128 countries were at risk of contracting dengue. It has been estimated that the disease affects 824 million urban households and 763 million peri-urban homes [3].

There are four distinct dengue virus serotypes (DENV1-4), all of which originate from the family *Flaviviridae* and genus *Flavivirus*. The fifth and latest proposed serotype of the dengue virus is DENV5, announced in October 2013. DENV5 was detected during the screening of viral samples taken from a patient admitted in a hospital in the Malaysian state of Sarawak in the year 2007. Genetic recombination, natural selection, and genetic bottlenecks have been implicated as factors that can lead to the emergence of new serotypes [4].

The viral infection may be eventually asymptomatic, but may also result in disease, ranging from rapid undifferentiated febrile episodes to severe life-threatening forms. The virulence is associated with a serotype and its genetic variations. Secondary infections are related to the occurrence of the most severe forms of the disease—dengue hemorrhagic fever (DHF) and dengue shock syndrome (DSS)—however, DHF/DSS can also occur in primary infections [5].

In the most acute infection models, neutralizing and non-neutralizing antibodies are involved in viral control, elimination, and protection. However, a possible harmful role of virus-specific antibodies has been described. It has been pointed out that they may be the cause of worsening of infection in in vitro assays. This occurs by a mechanism called antibody-dependent enhancement (ADE) that occurs when pre-existing antibodies present in the body from a first dengue infection bind to another infecting DENV particle during a subsequent infection. The primary infection antibodies might not neutralize the virus. Instead, the virus-antibody complex binds to the Fcy receptor in the circulating monocytes. The antibodies help the virus to infect the monocytes more efficiently. The result is an overall increase in virus replication and increased risk of more severe dengue forms [6].

Treatment of dengue is symptomatic as no specific antiviral drugs are available, therefore, the WHO considers the establishment of an effective vaccine against these viruses a priority. The ideal dengue vaccine must have low levels of side effects, providing lasting protection and be tetravalent to prevent the occurrence of ADE. As the regions where dengue occurs are underdeveloped, the vaccine must be economically viable and preferably be administered in a single dose [7]. Research groups throughout the world have been trying to develop a vaccine against dengue, using some classical methods, such as, viral attenuation until the recombinant antigens are expressed into the expression vectors [8–17].

Efforts to create a dengue vaccine by conventional techniques such as attenuation and virus inactivation were not efficient. For this, molecular biological methods have also been employed to search for new strategies to develop vaccines against dengue, ranging from the viral chimeras to the development of DNA vaccines [11, 12, 18–21]. The latter consists of a recombinant plasmid optimized for expression in eukaryotic cells encoding for expression of the antigen of interest [22].

DNA nuclear import is a relatively inefficient process, mainly due to the presence of cytoplasmic nucleases that can degrade the DNA plasmid before it reaches the nucleus [23]. Therefore, the use of new methods to improve the DNA nuclear import process is necessary as a fundamental part of the advancement in the DNA vaccine research. The use of carbon nanotubes (CNT) for the introduction of exogenous DNA into cells is a great interest area today and is in an intense phase of expansion. Studies must be carried out to better understand how to use them and the potential risks involved in its animals use. Determination of optimal conditions for transfection, cell internalization mechanisms, routes of inoculation into animals, the role of electrical charge density, as well as significance of the length and width of the CNTs in transfection efficiency, need to be elucidated with additional studies.

The CNTs are categorized into two main types: single-walled carbon nanotubes (SWCNTs) and multi-walled carbon nanotubes (MWCNTs). Their lengths can vary from a few 100 nm to a few microns and their diameter varies between 0.4–2 nm and 2–100 nm for SWCNTs and MWCNTs, respectively [24]. They are composed exclusively of carbon atoms, which are arranged in a regular hexagonal pattern in the shape of benzene rings forming graphene sheets [25].

In recent years, studies on CNTs have demonstrated that there may be an efficient way of increasing the internalization as also the expression of DNA sequences, such as, vector plasmid expression [26–29]. However, these studies are very recent and little is known about using CNTs and how to improve their efficiency. Further investigation needs to be done in this area that is booming today.

As there are few studies evaluating the CNTs behavior, such as, DNA vector in Vero cells and in vivo by the intramuscular route and as none of these studies used MWCNTs, this study aimed to assess the efficiency of MWCNTs in the introduction of exogenous DNA in vitro and in vivo.

## Results and discussion

### Conjugation of DNA to the CNTs and ability of CNTs to reach the cellular interior

There is an increasing demand for delivery of biological materials into cells, as success in various therapies has been limited by the difficulty in making these materials reach the cells’ interior. The current methods used to introduce pure DNA into cells, combine low efficiency with significant cellular toxicity [30].

For plasmid DNA conjugation with the CNTs, both were subjected to sonication together. Subsequently transmission electron microscopy (TEM) was performed in four different groups: pure MWCNT, MWCNT-DNA, pure c-MWCNT and DNA-c-MWCNT (c-MWCNT indicates that the MWCNTs were carboxylated).

**Fig. 1:**
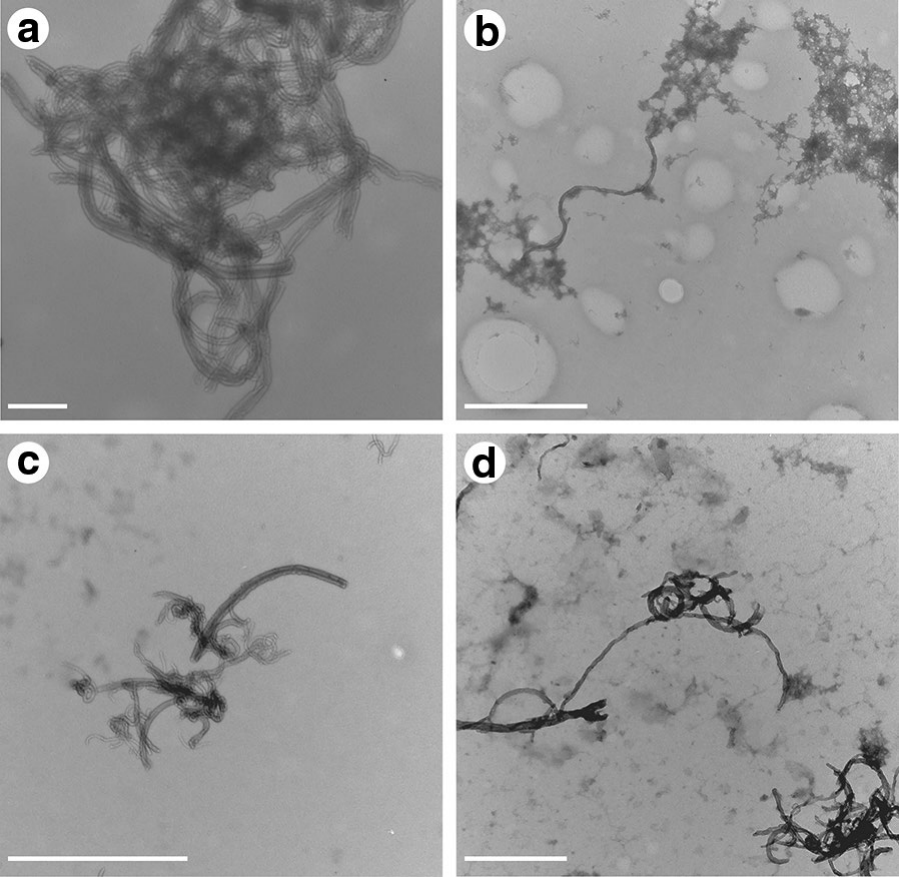
TEM of MWCNTs combined or not with plasmid DNA. TEM of MWCNT pure and combined with plasmid DNA. **a** MWCNT; **b** DNA-MWCNT; **c** *c*-MWCNT; **d** DNA- *c*-MWCNT. *Scale bars* in **a** indicate 200 nm; in **b**, **c** 1 μm and in **d** 500 nm

Transmission electron microscopy showed the functionalization of CNTs with the plasmid DNA and that c-MWCNTs had the same affinity for DNA than the MWCNTs not carboxylated. TEM also showed that the DNA had preferably connected at the ends of the both nanotubes types, with a higher tendency to form large clumps (Fig. 1).

**Fig. 2:**
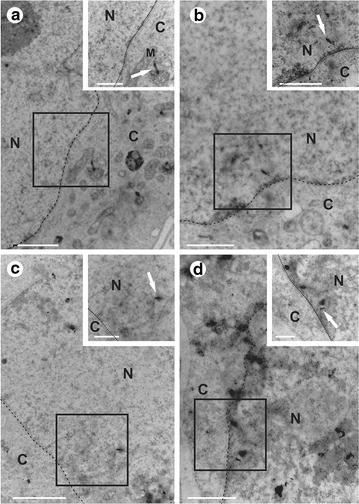
TEM of Vero cells transfected with MWCNTs. TEM of Vero cells transfected with MWCNTs (*arrows*) (**a**) and (**b**) *c*-MWCNT (**c**) and (**d**). **a**, **b** and **c**, **d** show that they are in the cytoplasm and core, respectively. Details highlighted in the *upper box*. The *scale bar* indicates 2 μm in **a** (1 μm) and **C** (1 μm); 1 μm in **b** (1 μm) and **d** (500 nm). Details are indicated between *parentheses*. *N* core, *C* cytoplasm, *M* mitochondria

To verify if the CNTs were able to reach the cell interior, Vero cells were transfected with the MWCNTs and c-MWCNTs and were subsequently subjected to TEM to check for the presence of CNTs inside the cells. The results showed that the CNTs had entered the cells, and were located in the cytoplasm and nucleus. However, the cells transfected with c-MWCNTs had a greater tendency to introduce CNTs within the nucleus (Fig. 2).

In recent times, studies have demonstrated that carbon nanotubes are good candidates for use as vehicles for transfection of exogenous material into cells, such as DNA and proteins, as they cross the cell membrane in a passive manner, without causing damage to the cell and they have low cytotoxicity levels. As seen in the studies of Bianco and Kostarelos, nanotubes have been internalized by Vero cells and are found in the cytoplasm and nucleus [29, 31]. It has been seen that there is an association of plasmid DNA with both carboxylated MWCNTs, and it is non-carboxylated. These results are highly encouraging, with the use of carbon nanotubes as DNA vectors.

### Real-time PCR of transfected cells

**Fig. 3:**
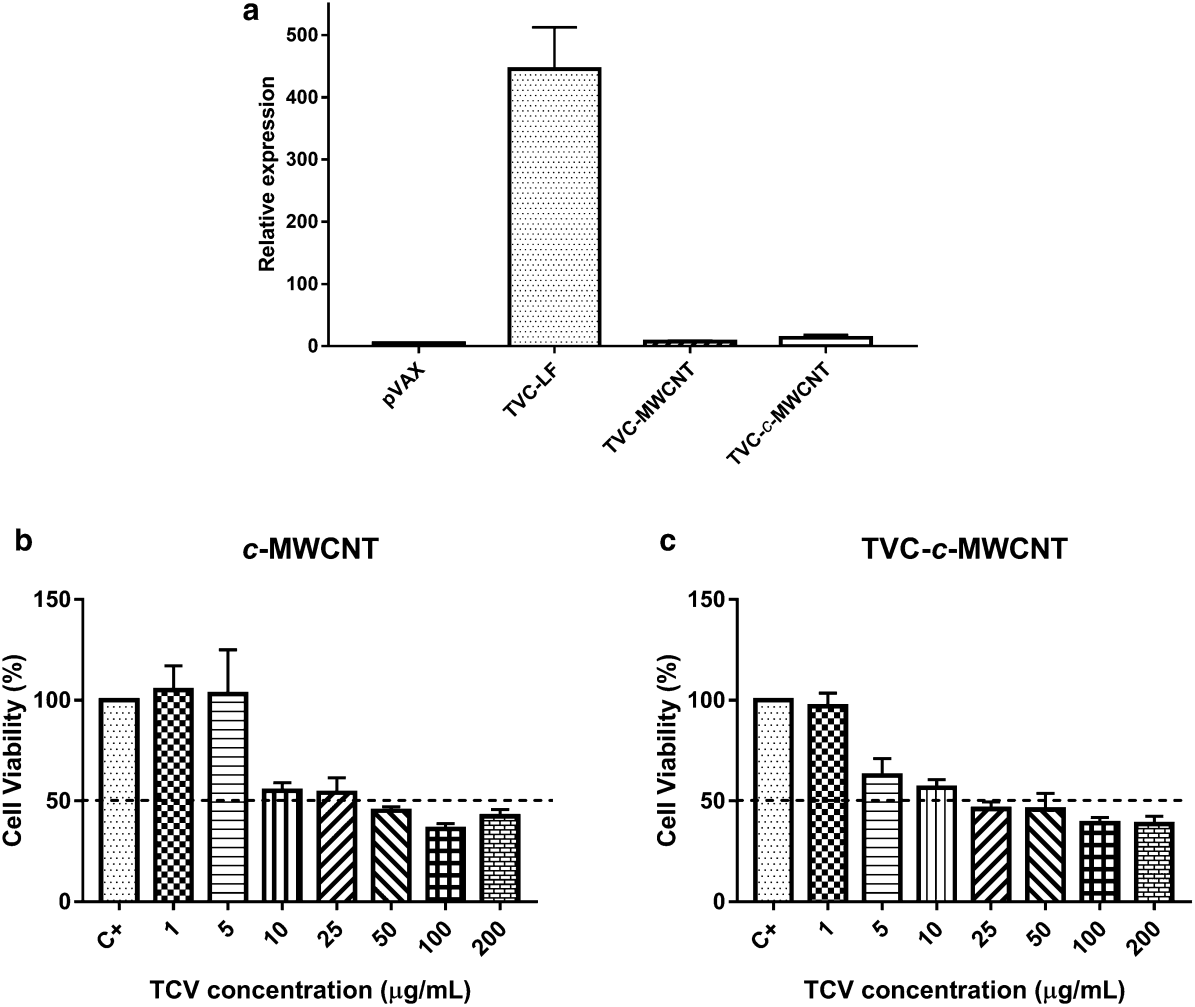
RT-PCR of Vero cells transfected and MTT assay. **a** RT-PCR of Vero cells transfected with the empty pVAX plasmid, only TVC, MWCNT functionalized with TVC, and *c*-MWCNT functionalized with TVC. **b**, **c** MTT assay in Vero cells treated with *c*-MWCNTs pure preparations (*c*-MWCNT) (**b**) or functionalized with plasmid DNA (TVC-*c*-MWCNT) (**c**) to evaluate the cytotoxicity of preparations

Cells transfected with carboxylated and not carboxylated MWCNTs, when used alone or combined with plasmid DNA, were analyzed, to see if the TVC conjunction with MWCNTs led the cells to produce RNA messengers of domain III of the E protein. For this, a real-time polymerase chain reaction (PCR) was carried out, using the primers for domain III of the E protein of DENV2. It showed that only cells transfected with TVC using Lipofectamine^®^ 2000 had a significant increase in the mRNA levels, while the TVC in conjunction with MWCNTs and c-MWCNTs had a smaller increase in mRNA compared to the control, which was represented by cells transfected with the empty pVAX plasmid (Fig. 3).

The only study that used Vero cells demonstrated that a high concentration of SWCNTs had significant toxicity in this cell type, whereas, a low concentration of SWCNTs retained their potential as carriers for drug delivery [32]. Our results indicated that a low concentration of MWCNTs for introduction of exogenous DNA into Vero cells was not efficient, suggesting a different behavior for SWCNTs, when compared their efficiency to delivery.

### Western blot

**Fig. 4:**
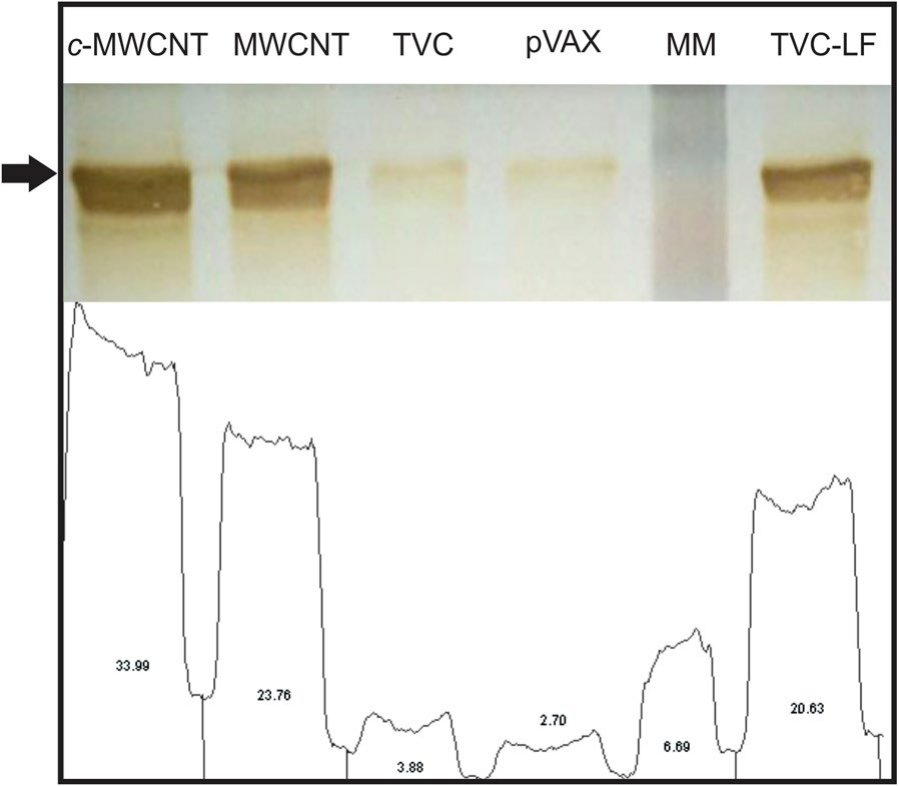
Western blot. Western blot and densitometry analysis of the different treatments. Were evaluated *c*-MWCNT and MWCNT functionalized with TVC, only TVC and TVC with Lipofectamine^®^ (TVC-LF) and empty pVAX. Densitometric analysis was performed using ImageJ software

In this assay is possible to confirm the great capability of MWCNT and the improved *c*-MWCNT, which shown densitometry values 10X higher when compared to the CVT alone and 63 e 48 % higher than the CVT transfected using as vehicle Lipofectamine^®^ 2000 and MWCNT, respectively (Fig. 4). This finding is surprising, in real time PCR was found that TVC-LF had a higher expression, but western blot assay shown that some few mRNA was translate into protein. Some factors are related to this finding, such as: transcription rate determined by mRNA sequence; transcription rate modulation, related to de elements involved with external elements (regulatory proteins and ncRNA, for example); protein stability; protein translate rate and protein transport [33]. Cheng et al. [34] observe that protein regulation system act as a commutation, when mRNA expression increase, this will be counterbalanced by translate protein regulation steps, to achieve a steady state. Theoretical studies demonstrate the stabilization of proteins in the presence of multiwalled nanotubes [35], particularly due to the electrostatic or hydrophobic interactions of certain protein regions and multiwalled carbon nanotubes.

### Cell viability assay with tetrazolium salt

**Fig. 5:**
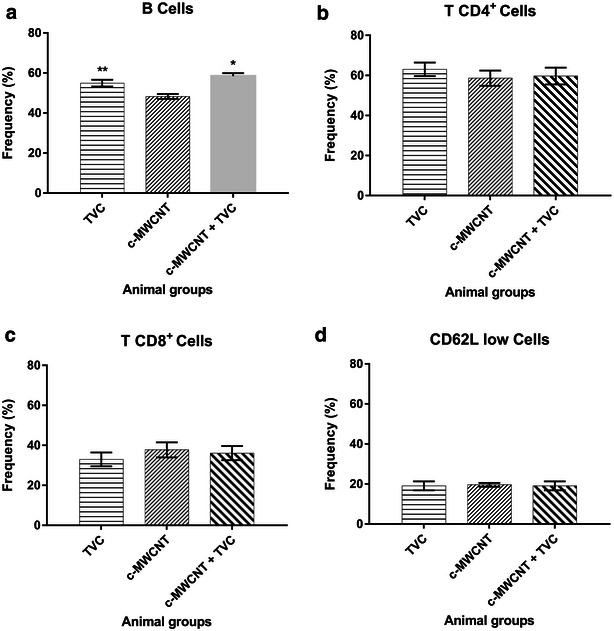
Immunophenotyping. Immunophenotyping of lymphocytes of animals subjected to four different treatments (pVAX, TVC, c-MWCNT, TVC-c-MWCNT). **a** B cells, **b** T CD4+ cells, **c** T CD8+ cells, **d** CD62L low cells

Before proceeding with studies in mice, we first made a cell viability MTT assay, to determine which concentration of CNT generated a cytotoxic effect in Vero cells. For this, the cells were incubated with four different preparations (MWCNT, DNA-MWCNT, c-MWCNT, and DNA-c-MWCNT) in increasing concentrations of CNTs (from 1 to 200 µg/mL). The data obtained from the c-MWCNTs were used as the basis for a choice of 10 µg/mL as the concentration used in further studies, as also the chosen concentration for the in vivo assay (Fig. 5b, c). The concentration was consistent with other studies, which showed that the CNT concentrations between 5 and 20 mg/mL had no cytotoxicity when tested in a cell culture [36, 37]. Adding DNA had little or no impact in the net toxicity of CNTs.

### Immunophenotyping, lymphoproliferation, and cytokine detection

**Fig. 6:**
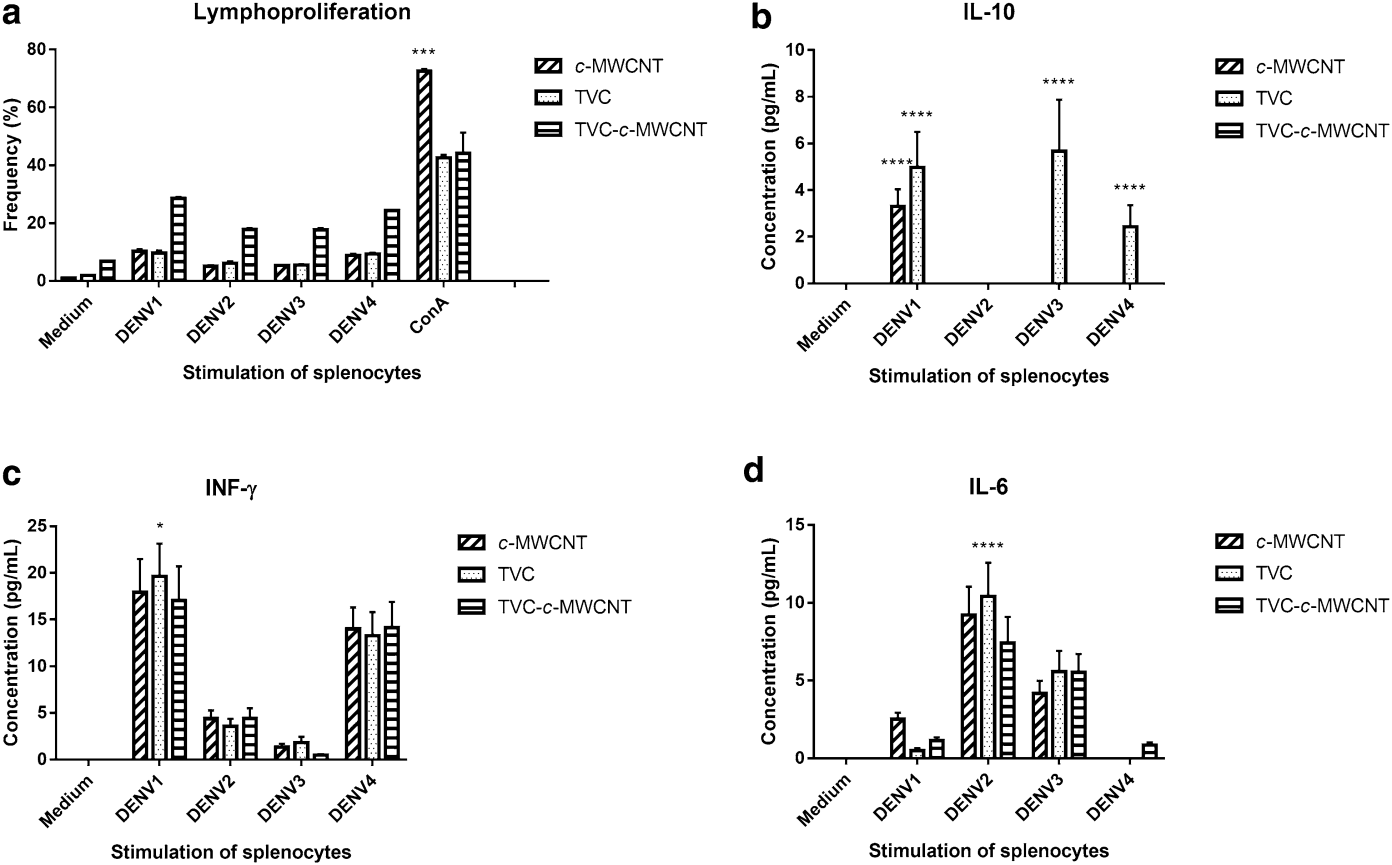
Lymphoproliferation and cytokine production. **a** Lymphoproliferation of lymphocytes of animals subjected to four different treatments (TVC, c-MWCNT, TVC-c-MWCNT) when stimulated with the four serotypes of dengue virus, the medium (negative control), and Concanavalin A (ConA) (positive control). **b**–**d** Featured cytokine that had a significant increase, showing the levels produced by lymphocytes of animals subjected to four different treatments (TVC, c-MWCNT, TVC-c-MWCNT) when stimulated with the four serotypes of the dengue virus and the medium (negative control)

In the final part of the study, tests were done to determine the immune response profile generated in mice subjected to four different treatments (empty plasmid-pVAX, tetravalent vaccine candidate-TVC, c-MWCNT, and TVC-c-MWCNT carboxylated). In the immunophenotyping assay, the number of B, CD4+, CD8+, and CD62L_low_ cells for individual animals of each treatment group was measured. When the TVC or TVC-c-MWCNT groups were compared with the pVAX group, only the B cells showed a statistically relevant increase in cell count (Fig. 6). A slight, but not statistically significant increase could be observed in the CD8+ cell count in the c-MWCNT group. In the lymphoproliferation assay it was not possible to observe a significant increase of lymphocytes among the groups of animals treated with TVC, c-MWCNT, TVC-c-MWCNT, and the pVAX group, under different stimuli, but the difference was very strong as compared to that found when TVC-c-MWNTC was compared with TVC or f-MWNTC (Fig. 6a). The increase in the production of B cells in animals treated with TVC or conjugated TVC-c-MWCNTs was an important finding, once it indicated an increase in antibody-producing cells in these animals—a desired characteristic to use as a future vaccine.

The production of cytokines by lymphocytes from animals when stimulated with the four serotypes of virus, in the four different treatments (pVAX, TVC, c-MWCNT and TVC-c-MWCNT). A significant increase in IL-10 production has been observed in animals treated with TVC alone, while IL-6 and IFN-γ have different behaviors, being IL-6-induced by DENV serotypes 2 and 3 and INF-induced by serotypes 1 and 4, for all groups, including the pVAX group. A difference in production of these cytokines has not been observed when TVC has been administered in conjunction with c-MWCNT (Fig. 6b–d). The production of IFN-γ and IL-6 is significant for a response against a virus, as these are pro-inflammatory cytokines that assist in fighting infection. IL-10 production is not very favorable to an anti-viral response-frame, as this is an anti-inflammatory cytokine that inhibits the immune response cells, such as, macrophages and dendritic cells, and is seen only in the TVC group. However, this can signal the Th2 pattern, once when IL-10 is produced in conjunction with IL-4 and IL-12 induces Th2 response.

Perhaps this weak immune response produced by animals treated with TVC conjugated with c-MWCNT could be because of the immunization route chosen, which was intramuscular, in contrast to the previous studies analysing the effect of CNTs on animals, which used intravenous [27, 38–40], intraperitoneal [41, 42], and subcutaneous [43] routes, with formation and generation of a more intense immune response at the site of the inoculum. The few studies that used the intramuscular route for viral immunization with CNTs worked with fish and used SWCNTs [44–46].

## Conclusions

This study showed that multi-walled carbon nanotubes (carboxylated or not) were internalized by Vero cells and were found in the cytoplasm and nucleus, associated with plasmids. We had to use low concentrations of MWCNTs for introduction of exogenous DNA into Vero cells and it was not efficient to increase the mRNA compared to the TVC transfected with Lipofectamine^®^ 2000 (TVC-LF), however, it was possible to observe that c-MWCNT had a higher expression level than the non-carboxylated MWNTC and protein production was higher than both. This result suggested a similar behavior that was reported for SWCNTs. Carbon nanotubes showed great potential for immune response modulation to Th2 pattern with an increasing of B cells, interesting behavior for the vaccines development with the antibodies production. However, other studies could be conducted to enhance the immune response, improve Th1 pattern and achieve the same to all serotypes, once this study shown a different responses, with DENV1 and DENV4 had the same pattern, like as DENV2 and DENV3, what suggest a relationship with the position of each epitope in the construction, once the secondary structure it is one of the antigenicity and specificity determinants [47]. Beside preliminary studies shown reduction in viral titer to some serotypes DENV1 and DENV2 (Additional file 1: Figure S1), more studies will be done to better immune response, especially to improve Th1 pattern and use others cell types to verify the influence in the results found in this work.

With regard to the in vivo use of an intramuscular injection, the combination of our vaccine candidate with MWCNTs reduced the immune response when compared to TVC. This result discouraged use of the intramuscular route, to verify if the MWCNTs were good as biological material vehicles for viral immunization in mammals. However, it would be interesting to have studies comparing various routes of inoculation, to confirm this result. Therefore, studies conducted with nanoparticles as biological material vehicles should be encouraged.

## Methods

### Cell culture

Vero cells (from monkey kidney) were maintained at 37 °C, in an atmosphere of 5 % CO_2_, in minimal essential medium eagle (MEM) supplemented with 10 % fetal bovine serum, for promoting growth and 2 % for maintenance of the cell line, in addition to antibiotic solutions: penicillin (500 U/mL) and streptomycin (100 µg/mL).

### Viral stock

The dengue viruses used in this study were the following strains: DENV1 (Nauru Island), DENV2 (New Guinea C), DENV3 (H87), and DENV4 (H241), obtained from the viral stock of the Molecular Immunovirology Laboratory of the Federal University of Viçosa. Samples from each serotype were inoculated into the brains of newborn Swiss mice. After the onset of viremia, the brains were removed, macerated with 1X PBS (phosphate-buffered saline) and subjected to centrifugation at 2500×*g* for 10 min. Next, the supernatant was supplemented with fetal bovine serum to a final concentration of 20 %, aliquoted, and stored in a freezer at −80 °C.

### DNA vaccine candidate tetravalent

The TVC used was produced in the Molecular Immunovirology Laboratory of the Federal University of Viçosa. It was composed of the optimized plasmid vector for expression in mammalian cells pVAX1© (Invitrogen Corporation, California—USA), which had inserted genes of Domain III of E protein serotypes DENV1, DENV2, DENV3 and DENV4, forming a tandem sequence of these four gene fragments, generating a polypeptide of 60 kDa. This construction was confirmed by PCR, restriction, and sequencing assays.

### Multi-walled carbon nanotubes

The MWCNTs used in this study were obtained from the Nanomaterials Laboratory of the Physics Department of the Federal University of Minas Gerais, granted by Professor Luiz Orlando Ladeira. These nanotubes were produced by the chemical vapor deposition process from a carbon precursor (ethylene) and a base catalyst to nanostructured metal particles (Ni, Fe, Co) having a purity level that was in the order of 95 %. The carboxylation of the nanotubes was made via using a microwave in an acidic medium (nitric/sulfuric). It first generated defects in the structure and subsequently stabilized these defects with the carboxylic groups and, the degree of carboxylation was in the order of 10 %.

### Synthesis of DNA-MWCNT

In conjunction with the TVC MWCNT, 20 µg of plasmid DNA were placed in PBS solution with carbon nanotubes, at a concentration of 10 µg/mL. This solution was sonicated for 30 min in an ultrasonic bath, to enable the conjugation of DNA to the wall of the MWCNT and this was kept on ice until use.

### Transmission electron microscopy of DNA-MWCNT

To characterize the DNA-MWCNT four different samples of MET were made: pure MWCNT, MWCNT-DNA, pure c-MWCNT, and DNA-c-MWCNT. After the process of sonication for 30 min, the four samples were separately placed on and covered with formvar grids for 1 min and 30 s. Subsequently they were dried and aqueous 0.5 % uranyl acetate was added for 15 s and dried, to make the contrast of DNA. The samples were left in the desiccator for 12 h and visualized in the electron microscope Zeiss transmission EM 109, which belonged to the Center for Microscopy and Microanalysis, at the Federal University of Viçosa.

### Transfection of vero cells

For transfection of cells with pure plasmid DNA (both empty plasmids, as TVC), we used the protocol with Lipofectamine^®^ 2000 transfection reagent (invitrogen) in which 100 µL of MEM medium, without fetal bovine serum, was added to the DNA plasmid in a tube. In another tube, 1 µL of Lipofectamine^®^ 2000 was added for 1 µg of DNA in 100 µL of MEM medium. It was left for 5 min at room temperature. Both tubes were mixed and left for 20 min at room temperature. Thereafter, 200 µL of this mixture was added to the cells and incubated for 24 h. For transfection of cells with MWCNT, they were used at a concentration of 10 µg/mL and sonicated for 30 min with and without the plasmid DNA (20 µg) and added directly to the cell culture medium and incubated for 24 h.

### Extraction of total RNA from cells and preparation of complementary DNA

After the cells were incubated for 24 h, they were collected and total RNA extraction was done using the RNA Isolation PureZOL Reagent (Bio Rad) according to the manufacturer’s recommendations. This total RNA was used for making the complementary DNA (cDNA) using random primers and the reverse transcription reagent GoScript Reverse Transcriptase (Promega), following the manufacturer’s recommendations.

### Western blot

Domain III expression from Vero cells was evaluated, the different cell extracts (pVAX, TVC, MWCNT, c-MWCNT and TVC transfected using Lipofectamine^®^ 2000) precipitated with TCA were applied on polyacrylamide gel 10 %, transferred to a nitrocellulose membrane using a Semi-dry electrotransfer mini Trans Blot Cell (Bio-Rad, California, USA) and transfer buffer (25 mM Tris, 190 mM glycine and 20 % methanol). After 1 h transfer membranes were left in blocking solution (PBS, 0.5 % Tween 20 and 5 % dried milk) for 16 h under mild stirring. Later, dengue virus polyclonal antibodies (1:3000) produced in mice were added to the blocking solution. Antibodies were added and left in contact with the membranes for 4 h. The samples were then washed with PBS-Tween three times for 5 min and secondary antibody (1:10,000) anti mouse IgG conjugated to peroxidase was added to membrane and left in contact with the membrane for 1 h. After the membranes were washed with PBS-Tween three times for 5 min and revelation was performed using DAB (3,3′-diaminobenzidine) is dissolved in 50 mM Tris–HCl (pH 7.6). Forty microliter of hydrogen peroxide were added to each membrane and left for a few minutes, and the protein bands appear on the membrane the reaction was quenched with distilled water and the membranes were allowed to dry and photographed. The bands densitometry analysis was performed using ImageJ software (https://www.imagej.nih.gov/).

### Real-time polymerase chain reaction

For real-time PCR, specific primers of DENV-2 domain III were used at a concentration of 200 nM and 400 ng of cDNA transfected cells with four different treatments: pVAX, TVC, TVC-MWCNT and TVC-c-MWCNT. We used the Eco Real-Time PCR System (Illumina) and the data have been analyzed in the EcoStudy software (Illumina). The amplification profile was: 50 °C for 2 min, 95 °C for 10 min, and 40 cycles at 95 °C for 10 s, 60 °C for 30 s, and 95 °C for 15 s. The primers were 5′CCCCTTAAGGTGCAGGCTGAGAATGGACA3′, and 5′GGGGGTACCTCGCCCAAAATGGCCATTC3′, generating a fragment of 417 bp.

### Transmission electron microscopy of vero cells

To view the nanotubes within the Vero cells, they were incubated with MWCNTs c-MWCNTs at a concentration of 10 mg/mL for 3 h, and subsequently, the MEM was removed and trypsin added to release the culture flask of cells. The cell suspension was collected, centrifuged at 1500×*g* for 10 min and the supernatant was discarded. The cell pellet was washed thrice with PBS at 1500×*g* for 5 min, and after washing, the cells were fixed for 2 h in a fixative solution (2.5 % glutaraldehyde, 0.1 M sodium cacodylate, sucrose 0.2 M). The fixed cells were washed with 0.1 M sodium cacodylate for 5 min and post-fixed in osmium tetroxide for 30 min. To remove the excess osmium, the cells were washed with 0.1 M sodium cacodylate for 5 min and then dewatering steps were carried out with solutions of increasing concentrations of ethanol (50, 70, 90, 95 and 100 %). Subsequently, the cells were infiltrated with LR White resin (Agar Scientific) in a ratio of 2:1 (100 % alcohol:resin) for 40 min, then with resin LR White (Agar Scientific) in a ratio of 1: 1 (100 % alcohol:resin) for 40 min, and finally, infiltrated with pure LR White resin for 16 h and left. All these resin infiltration steps were done in a refrigerator at 4 °C. After this time the resin was removed and placed in new neat resin sample tubes themselves to the biological material, leaving them in an oven at 65 °C for 48 h to solidify the resin. At the end of this time the resin blocks with the biological material were removed from the tubing and taken to be made into ultramicrotome material sections (100 nm), which were collected on grids and then counterstained. For this step, the grids were allowed to soak in 1 % uranyl solution for 15 min, washed 50 times in distilled water, left in lead citrate solution for 8 min and finally washed more than 50 times in distilled water. The grids were put under the transmission electron microscope to obtain images. This was carried out in the Center for Microscopy and Microanalysis at the Federal University of Viçosa.

### Evaluation of cell viability by a colorimetric test using MTT

In order to assess whether the MWCNTs were toxic to Vero cells was done cell cytotoxicity assay with the same, which were grown in 96 well plates with an initial number of 104 cells per well, to reach confluency. Cells were grown in MEM medium supplemented with 10 % fetal bovine serum at 37 °C in an atmosphere of 5 % CO2. After adhesion of the cells, they were removed from the medium and added to 100 µL of MWCNT, DNA MWCNT c-MWCNT and DNA-c-MWCNT samples, diluted in MEM medium, supplemented with 2 % fetal bovine serum, at concentrations of 1, 5 10, 25, 50, 100, and 200/mL, in nanotubes. Control of this test was done with pits which were only added MEM and all the tests were done in triplicate. Subsequently, the plates were incubated for 24 h, after which time the medium was removed and 100 µL of MTT (5 mg/mL) diluted tenfold in MEM medium and supplemented with 2 % fetal bovine serum was added to the cells in all wells and the plates were incubated for 4 h under the same conditions. After incubation, the MTT-containing medium was removed and 100 µL of isopropanol-HCl solution was added to the wells, to solubilize the formazan crystals. The plates were shaken gently at room temperature for 20 min for solubilization of the crystals and a reading was done on a spectrophotometer with a wavelength of 590 nm.

### Immunization of mice

In this study, we used 5-week-old BALB/c female mice. They were divided into four groups, with four animals in each group, according to the inoculated material in them. The groups were: Group 1: pVAX; Group 2: TVC; Group 3: c-MWCNT; Group 4: TVC-c-MWCNT. For tests with these mice, 100 µg of DNA and nanotubes at a concentration of 10 µg/mL were used, and this step, in vivo studies on the c-MWCNTs, was used only once. The non-carboxylated CNTs were insoluble and generated a greater local inflammatory response. A day prior to the first immunization dose of some mice, blood was collected by venipuncture in a retro-orbital bag and processed to obtain the serum, which was called the pre-immune serum. In this procedure, blood samples were left at room temperature for 30 min to allow blood clotting, then centrifuged at 2500×*g* for 10 min and the serum contained in the supernatant was collected and pooled into a single serum pool, which was stored at −20 °C. The following day, each mouse from each of the four groups was inoculated intramuscularly with the samples belonging to each group. These immunization procedures were repeated twice more, with an interval of 15 days between them. Furthermore, 14 days after each of the three immunizations, blood from each mouse was collected and processed to obtain the serum, just as was done with the pre-immune serum, and the serum pools collected from each group were stored at −20 °C and subsequently tested for the presence of antibodies against different serotypes of dengue viruses. The mice were provided by the Central Animal Facility of the Federal University of Viçosa (UFV) and kept in the vivarium at the Molecular Immunovirology Laboratory of UFV. Throughout the experimental period the animals had free access to water and food and were kept in room temperature, controlled ventilation and photoperiod, and the methodology used in this project was approved by the Ethics Committee on Animal Use of the Federal University of Viçosa. The process was named 70/2013.

### Immunophenotyping

Spleens from each mouse immunized in the four different groups were obtained and macerated in RPMI with 2 % fetal bovine serum (FBS). The tissue extract was transferred to 15 mL conical tubes and centrifuged at 500×*g* for 5 min at 4 °C. The supernatant was discarded and the precipitate was suspended in an erythrocyte lysis buffer, left for 5 min at 4 °C and centrifuged again at 500×*g* for 5 min at 4 °C. Next, the supernatant was discarded and the precipitate was washed with RPMI with 2 % FBS and then suspended in the same medium. Cells were counted in a Neubauer chamber under an optical microscope and a calculation was made to use 1 × 107 cells per well. These were seeded into 96 well plates and added in the middle of an FACS buffer (1X PBS, FCS 2 %, and 3 mM EDTA). The cells were centrifuged at 500×*g* for 2 min in a plate centrifuge and then the supernatant was discarded and the antibody-specific precipitates were added to the lymphocyte surface molecules, which are conjugated antibodies with fluorophores, for detection by flow cytometry (CD3—FITC; CD4—APC Cy 7; CD8—PE Cy 7; CD19—PerCP Cy 5.5; CD44—PE; CD62L—PCA). They were left in contact with the cells for 30 min at 4 °C with an FACS buffer, then centrifuged at 500×*g* for 2 min and washed with the FACS buffer. The supernatant was discarded and the cells were suspended in the FACS buffer, to which was added propidium iodide (0.5/mL). Finally, the cells were taken for analysis in a cytometer, BD FACSVerse Flow Cytometer, from the Center for Microscopy and Microanalysis at the Federal University of Viçosa.

### Lymphoproliferation assay

Spleen cells collected during the methodology described in the previous section were counted in a Neubauer chamber and a calculation was made to use 1 × 107 cells per well. These were seeded in 96-well plates and a medium containing sterile PBS 1X, 0.1 % FBS, and CFSE dilution of 1: 1000 was added. The cells were left in this medium for 10 min at 37 °C, washed thrice with RPMI + FCS 10 %, centrifuged at 500×*g* for 5 min at 4 °C, and then the supernatant was discarded and the precipitate was suspended in RPMI medium with 10 % FBS. This means that the stimuli that had come from brain extracts of infected mice were added to samples of each of the four serotypes of dengue viruses (MOI of 0.1). The negative control cells were only used with the medium, and the positive control cells used were stimulated with Concanavalin A (7.5 mg/mL), and all tests were done in duplicate. After 3 days of stimulation, the cell culture supernatant was collected and stored in a freezer at −80 °C for use in the cytokine detection assay. The cell precipitate was added to the antibodies to CD4, CD8 and CD19 diluted in FACS buffer (1:400) and left in contact with them for 30 min at 4 °C. The cells were then washed thrice with FACS buffer 500×*g* for 2 min and suspended in the FACS buffer to which propidium iodide (0.5/mL) was added. This was brought in cytometer BD FACSVerse Flow Cytometer (BD) of the core for Microscopy microanalysis at the Federal University of Viçosa.

### Detection of cytokines

For detection of cytokines produced by the cells stimulated lymphoproliferation assay, the supernatant was collected and stored in a freezer at −80 °C, was analyzed using the BD CBA Mouse Th1/Th2/Th17 Cytokine Kit (BD), according to the recommendations of a manufacturer. It is possible to detect the cytokines IL-2, IL-4, IL-6, IFN-γ, TNF, IL-17A and IL-10 by using this kit. Samples were analyzed in the cytometer BD Flow Cytometer FACSVerse (BD) from the Center for Microscopy and Microanalysis at the Federal University of Viçosa.

### Additional file

**Additional file 1: Figure S1.** PRNT assay. Lysis plates reduction by neutralizing antibodies are evaluated by PRNT assay. Reduction was observed in two serotypes analyzed, dengue virus serotype 1 and 2 (DENV1 and DENV2).

## Abbreviations

WHO: World Health Organization
DENV: dengue virus
DHF: dengue hemorrhagic fever
DSS: dengue shock syndrome
ADE: antibody-dependent enhancement
DNA: deoxyribonucleic acid
cDNA: complementary DNA
RNA: ribonucleic acid
mRNA: messenger RNA
TVC: tetravalent vaccinal candidate
CNT: carbon nanotubes
SWCNT: single-walled carbon nanotubes
MWCNT: multi-walled carbon nanotubes
c-MWCNT: multi-walled carbon nanotubes carboxylated
TEM: transmission electron microscopy
MTT: tetrazolium Salt
IL-6: interleukin 6
IL-10: interleukin 10
IL-17A: interleukin 17A
IFN-γ: interferon-gamma
TNF: tumor necrosis factor
MEM: minimal essential medium eagle
PBS: phosphate-buffered saline
PCR: polymerase chain reaction
