# Supplementary figures and images for: Multi-walled carbon nanotubes increase antibody-producing B cells in mice immunized with a tetravalent vaccine candidate for dengue virus
Source: J Nanobiotechnology. 2016 Jul 27;14:61. doi: 10.1186/s12951-016-0196-7 (PMC4964006; doi:10.1186/s12951-016-0196-7)

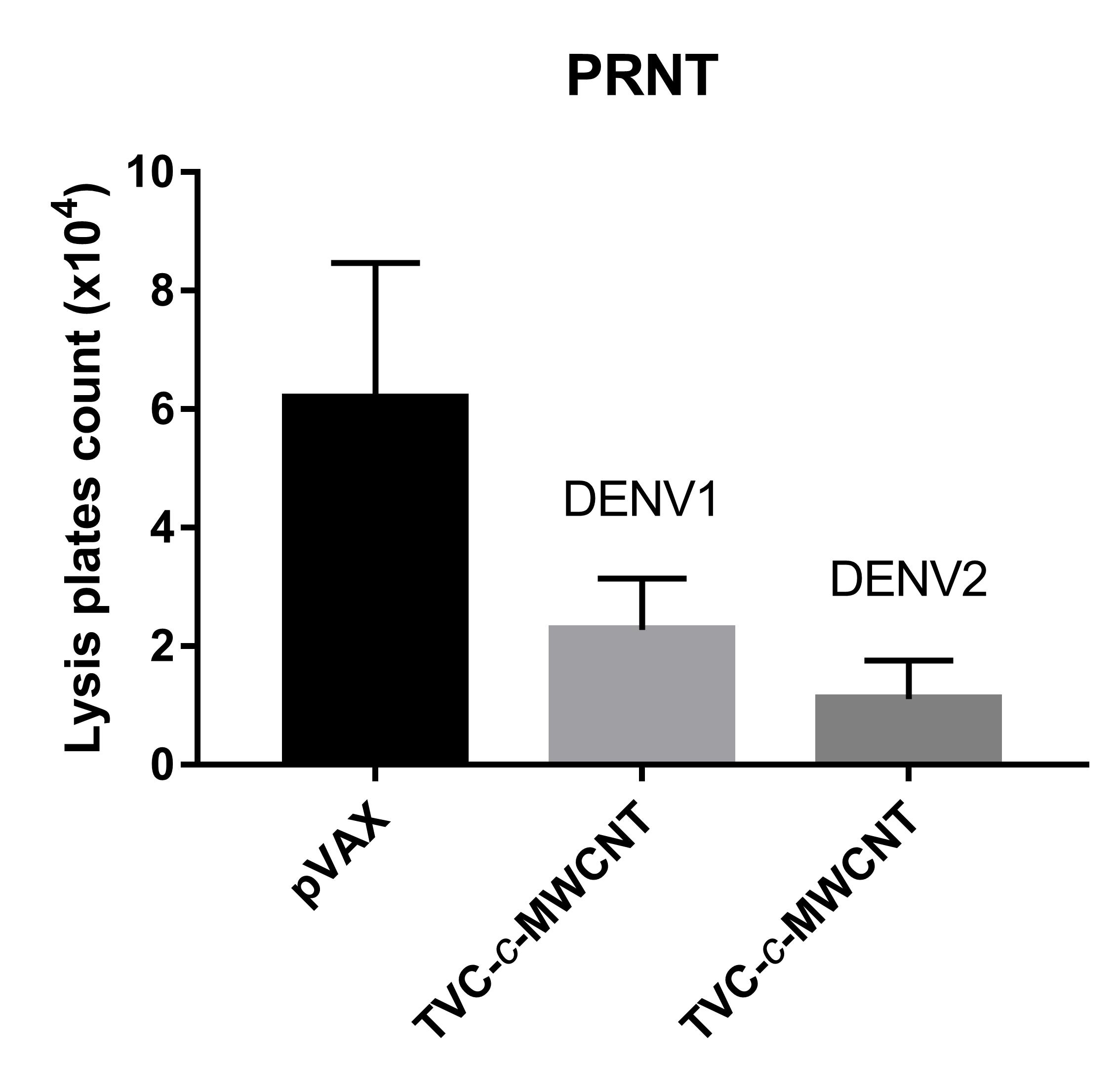

Supplement: Supplementary file 1 — Additional file 1: Figure S1. PRNT assay. Lysis plates reduction by neutralizing antibodies are evaluated by PRNT assay. Reduction was observed in two serotypes analyzed, dengue virus serotype 1 and 2 (DENV1 and DENV2). [file 12951_2016_196_MOESM1_ESM.jpg]
